# Dietary Yeast Hydrolysate Alters Serum Metabolic Profiles and Selected Fecal Bacterial Taxa in Lactating Dezhou Jennies

**DOI:** 10.3390/microorganisms14081768

**Published:** 2026-08-11

**Authors:** Zhangxinhan Wen, Jiaxin Liu, Zuowei Li, Tianzheng Wang, Pengshuai Li, Xinyi Mao, Yuhan Yin, Boying Dong, Yulong Feng, Honglei Qu, Qiugang Ma, Shimeng Huang

**Affiliations:** 1 State Key Laboratory of Animal Nutrition and Feeding, College of Animal Science and Technology, China Agricultural University, Beijing 100193, China; zl_one@163.com (Z.W.); jxliu@cau.edu.cn (J.L.); 15684302290@163.com (Z.L.); xiaogenishi@163.com (T.W.); lpsmyj@cau.edu.cn (P.L.); mxycindy0929@163.com (X.M.); vvvirrgil@outlook.com (Y.Y.); 15315788979@163.com (B.D.); 1688308@163.com (Y.F.); leihong_qu@163.com (H.Q.); maqiugang@cau.edu.cn (Q.M.); 2 Laboratory of Feed Grain Safety and Healthy Poultry Farming, Beijing Jingwa Agricultural Science and Technology Innovation Center, Beijing 101206, China; 3 Hebei Key Laboratory of Specialty Animal Germplasm Resources Exploration and Innovation, College of Animal Science and Technology, Hebei Normal University of Science and Technology, Qinhuangdao 066004, China

**Keywords:** yeast hydrolysate, lactating Dezhou jennies, serum biochemical parameters, serum metabolomics, fecal microbiota, nutrient metabolism

## Abstract

Yeast hydrolysate (YH) is a yeast-derived functional feed ingredient containing bioactive peptides, amino acids, nucleotides, β-glucans, and mannan oligosaccharides, which may exert prebiotic-like effects by regulating nutrient metabolism, host health, and intestinal microbial homeostasis. However, its effects on lactating Dezhou jennies remain largely unclear. This study investigated the effects of dietary YH supplementation on body weight change, serum biochemical parameters, serum metabolomic profiles, and fecal microbiota composition in lactating Dezhou jennies. Sixteen healthy lactating Dezhou jennies were randomly assigned to a control group fed a basal diet (MCON, *n* = 8) or a YH supplementation group fed the basal diet supplemented with YH (MYE, *n* = 8) for 60 days. Compared with the MCON group, the MYE group showed numerically higher final body weight and body weight change. Serum biochemical analysis showed that YH supplementation significantly decreased alanine aminotransferase (ALT) and aspartate aminotransferase (AST) activities and increased triglyceride (TG) concentrations, while all measured biochemical parameters remained within physiological reference ranges. Serum metabolomic profiling identified 193 differential metabolites between the two groups, including 55 upregulated and 138 downregulated metabolites in the MYE group. These metabolites were mainly associated with amino acid metabolism, lipid metabolism, bile acid metabolism, steroid hormone biosynthesis, mineral absorption, ABC transporters, and protein digestion and absorption, indicating that YH supplementation reshaped nutrient-related metabolic pathways in lactating Dezhou jennies. Fecal microbiota analysis showed no significant changes in α-diversity or overall community structure; however, the MYE group displayed numerically higher microbial richness indices, more unique ASVs and genera, a lower relative abundance of Bacteroidota, and a higher relative abundance of Bacillota. LEfSe analysis further revealed enrichment of specific bacterial taxa in the MYE group, including Christensenellaceae_R-7_group, *Anaerovorax*, and UCG-related taxa. Collectively, these preliminary findings suggest that dietary YH supplementation may alter selected serum biochemical indices and circulating metabolites and may selectively affect the relative abundance of certain fecal bacterial taxa in lactating Dezhou jennies. However, given the limited sample size, these results should be interpreted cautiously, and larger-scale studies are needed to confirm their reproducibility and practical significance.

## 1. Introduction

The Dezhou donkey (*Equus asinus*), one of the major indigenous donkey breeds in China, is valued for its large body size, adaptability, efficient utilization of fibrous feeds, and economic importance in livestock production [1,2]. As hindgut fermenters, donkeys possess distinctive digestive and metabolic characteristics that enable efficient use of roughage-based diets, while their nutritional and metabolic status remains highly responsive to dietary interventions [3,4]. Compared with other livestock species, however, nutritional regulation in donkeys has received relatively limited attention, particularly during physiologically demanding production stages. Lactation represents one of the most nutritionally challenging periods in female animals, during which energy and nutrient requirements increase substantially to support milk synthesis, maternal maintenance, and metabolic adaptation [5,6]. In lactating jennies, appropriate nutritional management is essential for maintaining body condition, metabolic homeostasis, milk production, and subsequent reproductive performance. Previous studies have shown that nutritional strategies during pregnancy and lactation influence maternal body weight, milk production, milk composition, and foal growth [7,8]. Therefore, optimizing nutritional management during lactation is important for improving maternal physiological status while ensuring adequate nutrient supply for suckling foals.

With the increasing restriction of antibiotic growth promoters, natural functional feed additives have attracted considerable attention as nutritional strategies to support animal health and productivity. Among these, yeast hydrolysate (YH), produced through enzymatic hydrolysis or autolysis of Saccharomyces cerevisiae, contains a variety of bioactive compounds, including peptides, amino acids, nucleotides, β-glucans, mannan oligosaccharides, B vitamins, and antioxidant-related substances [9,10,11]. Owing to its complex composition, YH has been associated with improvements in nutrient utilization, intestinal function, immune regulation, and antioxidant capacity in several livestock species. Previous studies have reported beneficial effects of YH or other yeast-derived products on growth performance, nutrient digestibility, intestinal morphology, immune responses, and gut microbial composition in pigs, poultry, and calves [12,13,14,15,16,17,18,19]. However, these responses vary according to animal species, physiological stage, dietary composition, and YH formulation, indicating that its biological effects are context-dependent. Despite growing evidence in other livestock species, studies investigating YH supplementation in donkeys remain scarce. In particular, little is known about the physiological and metabolic responses of lactating jennies to dietary YH supplementation. Lactation imposes substantial metabolic demands, yet the effects of YH on serum biochemical characteristics, systemic metabolism, and intestinal microbial composition during this period have not been comprehensively evaluated. Integrating serum biochemistry, untargeted metabolomics, and fecal microbiota analysis provides an opportunity to obtain a more comprehensive understanding of the physiological responses associated with nutritional intervention.

Therefore, the present study evaluated the effects of dietary YH supplementation on body weight change, serum biochemical parameters, serum metabolomic profiles, and fecal microbiota in lactating Dezhou jennies. We hypothesized that dietary YH supplementation would be associated with differences in selected physiological, metabolic, and microbial characteristics. By integrating serum biochemistry, untargeted metabolomics, and fecal microbiota profiling, this study provides a comprehensive assessment of the physiological responses to dietary YH supplementation and contributes to the development of evidence-based nutritional strategies for lactating donkeys.

## 2. Materials and Methods

### 2.1. Ethics Statement

All animal procedures were conducted in accordance with the guidelines for the care and use of experimental animals and were approved by the Animal Care and Use Committee of China Agricultural University (Approval No. AW90605202-1-05).

### 2.2. Animals, Experimental Design, and Dietary Treatments

A total of 16 healthy lactating Dezhou jennies (*Equus asinus*), aged 2–3 years and with a mean body weight of approximately 256.52 kg, were enrolled in the study. All animals were maintained under standardized husbandry conditions at Shandong Dong-E Ejiao Co., Ltd. (Liaocheng, China). The experiment followed a completely randomized single-factor design, with each jenny serving as an individual experimental unit. The animals were housed individually in semi-open pens and offered a farm-formulated basal diet ad libitum twice daily at 07:00 and 19:00, with free access to clean drinking water throughout the study. No probiotics or antibiotics were administered for at least three months before the trial or during the experimental period. Following a 3-day adaptation period to stabilize feed intake and acclimate the jennies to the experimental feeding procedures, the animals were weighed and randomly allocated to one of two dietary treatments (*n* = 8 per treatment). Before the start of the experiment, all jennies had been maintained under the same husbandry conditions, received the same basal diet, and had not been subjected to any previous dietary or management interventions. Therefore, the adaptation period was intended to minimize potential disturbances associated with the experimental protocol rather than to establish a new physiological or microbial baseline. The feeding trial lasted 60 days. Jennies in the control group (MCON) received the basal diet alone, whereas those in the yeast hydrolysate group (MYE) received the same basal diet (Table 1) supplemented with 0.5 g/kg yeast hydrolysate (DSM; Beijing DSM Biological Products Co., Ltd., Shanghai, China). According to the manufacturer, the yeast hydrolysate primarily contained amino acids and small peptides (approximately 30–50%) and nucleotides and their derivatives (approximately 5–15%), including 5′-inosine monophosphate and 5′-guanosine monophosphate. It also contained yeast cell wall-derived β-glucan and mannan oligosaccharides, together with B-group vitamins, trace minerals, glutathione, and RNA hydrolysates. The concentrations of individual amino acids, peptides, β-glucan, and mannan oligosaccharides were not independently determined in the present study. The product contained no viable yeast cells. Foals remained with their dams throughout the experiment to maintain routine lactation management but did not receive direct yeast hydrolysate supplementation. All foals were managed under the same husbandry conditions and had unrestricted access to suckle their dams throughout the study.

### 2.3. Body Weight Measurement and Sample Collection

Body weight was measured for each lactating jenny after a 12 h fasting period on the first and last days of the formal experimental period. Body weight change was calculated as the difference between final and initial body weight. At the end of the experimental period, blood samples were collected from each jenny via the anterior jugular vein into 5 mL sterile vacuum blood collection tubes. The samples were immediately centrifuged at 3000 rpm for 10 min to obtain serum. The upper serum layer was carefully transferred into sterile microcentrifuge tubes and stored at −80 °C until subsequent biochemical and metabolomic analyses. For fecal microbiota analysis, fresh fecal samples were collected from each jenny immediately after defecation using sterile rectal swabs. The samples were placed into sterile tubes, immediately frozen, and stored at −80 °C until microbial DNA extraction.

### 2.4. Serum Biochemical Analysis

Serum biochemical parameters were measured to evaluate the physiological and metabolic status of lactating Dezhou jennies in response to dietary yeast hydrolysate supplementation. The measured indices included total protein (TP), albumin (ALB), alanine aminotransferase (ALT), aspartate aminotransferase (AST), alkaline phosphatase (ALP), total cholesterol (TC), triglyceride (TG), high-density lipoprotein cholesterol (HDL-C), and low-density lipoprotein cholesterol (LDL-C). All serum biochemical indices were determined using commercial assay kits (Tianjin MicroNanoChip Co., Ltd., Tianjin, China) according to the manufacturer’s instructions. Measurements were performed using a fully automated biochemical analyzer (Pointcare V2, Tianjin MicroNanoChip Co., Ltd., Tianjin, China).

### 2.5. Serum Metabolomic Analysis

A 100 μL aliquot of each liquid sample was transferred into a 1.5 mL centrifuge tube, followed by the addition of 400 μL extraction solvent (acetonitrile–methanol, 1:1, *v*/*v*) containing 0.02 mg/mL L-2-chlorophenylalanine as the internal standard. The samples were vortex-mixed for 30 s and ultrasonicated for 30 min at 5 °C (40 kHz). Subsequently, the samples were incubated at −20 °C for 30 min to precipitate proteins and centrifuged at 13,000× *g* for 15 min at 4 °C. The supernatant was collected and evaporated to dryness under a gentle stream of nitrogen. The dried extracts were reconstituted in 100 μL acetonitrile–water (1:1, *v*/*v*), ultrasonicated for 5 min at 5 °C (40 kHz), and centrifuged again at 13,000× *g* for 10 min at 4 °C. The resulting supernatants were transferred into LC–MS sample vials for analysis.

To evaluate the stability and reproducibility of the analytical system, pooled quality control (QC) samples were prepared by mixing equal volumes of all study samples. QC samples were processed identically to the experimental samples and injected at regular intervals (every 5–15 injections) throughout the analytical sequence. LC–MS/MS analysis was performed using a SCIEX UPLC–TripleTOF 6600 system equipped with an ACQUITY UPLC HSS T3 column (100 × 2.1 mm, 1.8 μm; Waters, Milford, MA, USA). The mobile phases consisted of solvent A (0.1% formic acid in water–acetonitrile, 95:5, *v*/*v*) and solvent B (0.1% formic acid in acetonitrile–isopropanol–water, 47.5:47.5:5, *v*/*v*/*v*). The flow rate was 0.40 mL/min, the column temperature was maintained at 40 °C, and the injection volume was 2 μL. For positive ion mode, the gradient program was 0–3.0 min, 0–20% B; 3.0–4.5 min, 20–35% B; 4.5–5.0 min, 35–100% B; 5.0–6.3 min, 100% B; 6.3–6.4 min, 100–0% B; and 6.4–8.0 min, 0% B. For negative ion mode, the gradient program was 0–1.5 min, 0–5% B; 1.5–2.0 min, 5–10% B; 2.0–4.5 min, 10–30% B; 4.5–5.0 min, 30–100% B; 5.0–6.3 min, 100% B; 6.3–6.4 min, 100–0% B; and 6.4–8.0 min, 0% B. The UPLC system was coupled to a TripleTOF™ 6600+ quadrupole time-of-flight mass spectrometer (SCIEX, Framingham, MA, USA) equipped with an electrospray ionization (ESI) source operating in both positive and negative ion modes. The operating conditions were as follows: source temperature, 500 °C; curtain gas (CUR), 35 psi; ion source gas 1 (GS1), 50 psi; ion source gas 2 (GS2), 13 psi; ion spray voltage floating (ISVF), +5500 V in positive mode and −4500 V in negative mode; declustering potential, 80 V; and collision energy, 20, 40, and 60 eV (rolling CE). Data were acquired in information-dependent acquisition (IDA) mode over an m/z range of 50–1200.

### 2.6. Fecal Microbial DNA Extraction and PCR Amplification

Total microbial genomic DNA was extracted from fecal samples using the E.Z.N.A.^®^ Soil DNA Kit (Omega Bio-tek, Norcross, GA, USA) according to the manufacturer’s instructions. The integrity and concentration of the extracted DNA were assessed using 1.0% agarose gel electrophoresis and a NanoDrop^®^ ND-2000 spectrophotometer (Thermo Fisher Scientific, Waltham, MA, USA). DNA samples showing clear electrophoretic bands, an A260/A280 ratio of 1.8–2.0, and an A260/A230 ratio greater than 1.5 were retained for subsequent analysis. Purified DNA samples were stored at −80 °C until amplification. The V3–V4 hypervariable regions of the bacterial 16S rRNA gene were amplified using the primers 338F (5′-ACTCCTACGGGAGGCAGCAG-3′) and 806R (5′-GGACTACHVGGGTWTCTAAT-3′). PCR amplification was performed using an ABI GeneAmp^®^ 9700 thermal cycler (Applied Biosystems, Foster City, CA, USA). Each 20 μL reaction contained 4 μL of 5× FastPfu buffer, 2 μL of 2.5 mM dNTPs, 0.8 μL of each primer (5 μM), 0.4 μL of FastPfu DNA polymerase, approximately 10 ng of template DNA, and nuclease-free water. The amplification program consisted of an initial denaturation at 95 °C for 3 min, followed by 27 cycles of denaturation at 95 °C for 30 s, annealing at 55 °C for 30 s, and extension at 72 °C for 45 s, with a final extension at 72 °C for 10 min. Each sample was amplified in triplicate, and the replicate PCR products were combined. The amplification products were examined by 2.0% agarose gel electrophoresis. Target bands of approximately 500 bp were excised and purified using the E.Z.N.A.^®^ Gel Extraction Kit (Omega Bio-tek, Norcross, GA, USA).

### 2.7. 16S rRNA Gene Sequencing

Purified amplicons were quantified, pooled in equimolar amounts, and subjected to paired-end sequencing on an Illumina MiSeq PE300 platform according to standard protocols. Library preparation and sequencing were performed by Majorbio Bio-Pharm Technology Co., Ltd. (Shanghai, China).

### 2.8. Statistical Analysis

Each lactating jenny was considered an independent experimental unit, with eight experimental units per dietary treatment. Conventional physiological data, including body weight and serum biochemical variables, were analyzed using SPSS Statistics version 26.0 (IBM Corp., Armonk, NY, USA). Data are presented as the mean ± standard deviation. Normality was evaluated using the Shapiro–Wilk test, and homogeneity of variance was assessed using Levene’s test. Differences between the MCON and MYE groups were evaluated using an independent-samples Student’s *t*-test when the assumptions of normality and homogeneity of variance were satisfied. When these assumptions were not met, the Mann–Whitney U test was used. No missing values were imputed for conventional physiological outcomes, and observations were not excluded solely on the basis of statistical extremeness. Exclusion was considered only when a documented sampling, measurement, or technical error was identified. Statistical significance was defined as *p* < 0.05. Given the relatively small sample size, *p* values between 0.05 and 0.10 were not formally interpreted as statistical tendencies; any corresponding numerical differences were described cautiously without inferential claims.

Raw UHPLC–MS data were processed using Progenesis QI (Waters, Milford, MA, USA) for baseline correction, peak detection, peak integration, retention time alignment, and peak matching. Metabolites were annotated by comparison with the HMDB, METLIN, and the Majorbio in-house database (MJDB). Data preprocessing was performed on the Majorbio Cloud Platform. Metabolic features detected in at least 80% of samples within any group were retained. Missing values were imputed using the minimum observed value, followed by sum normalization. Variables with a relative standard deviation (RSD) >30% in QC samples were excluded, and the remaining data were log10-transformed. Principal component analysis (PCA) and orthogonal partial least squares-discriminant analysis (OPLS-DA) were performed using the R package ropls (version 1.6.2). Model robustness was evaluated using seven-fold cross-validation. Differential metabolites were identified based on a variable importance in projection (VIP) score > 1.0 and Student’s *t*-test (*p* < 0.05). KEGG pathway enrichment analysis was performed using the Python package scipy.stats (v1.0.0).

For fecal microbiota analysis, raw paired-end reads were quality-filtered using fastp version 0.19.6 and merged using FLASH version 1.2.7. Reads that failed to meet the quality criteria and chimeric sequences were removed. The remaining high-quality sequences were denoised using DADA2 to infer amplicon sequence variants. Representative ASV sequences were taxonomically classified using the RDP Classifier against the SILVA 16S rRNA reference database. The resulting taxonomic abundance table was used for all downstream analyses. Alternative classification algorithms available in the bioinformatics pipeline were not used to generate the final analytical dataset. To account for differences in sequencing depth, the ASV table was rarefied to the minimum number of valid sequences obtained among all samples before diversity and community-composition analyses. Rarefaction curves were examined to assess whether the sequencing depth adequately captured the major bacterial diversity in the fecal samples. Alpha-diversity indices, including ACE, Chao1, Shannon, and Sobs, were calculated using MOTHUR version 1.30.2. Because microbial diversity indices and relative-abundance data were not assumed to follow a normal distribution, differences in alpha diversity between the MCON and MYE groups were evaluated using the Mann–Whitney U test. Beta diversity was assessed by principal coordinate analysis based on Bray–Curtis dissimilarities using QIIME 2. Differences in overall fecal bacterial community composition between the two treatment groups were evaluated using permutational multivariate analysis of variance. Linear discriminant analysis effect size analysis was used to identify bacterial taxa with differential relative abundance between the MCON and MYE groups, using a linear discriminant analysis score threshold greater than 3.0. No multiple-testing correction was applied to the LEfSe results; therefore, the identified taxa were regarded as exploratory candidate discriminatory taxa requiring further validation. Because only fecal samples were collected, all microbiota findings were interpreted as reflecting fecal bacterial community composition. These data were not used to directly infer microbial functional activity, fermentation capacity, or the microbial characteristics of specific hindgut compartments.

## 3. Results

### 3.1. Effects of Dietary YH Supplementation on Body Weight Change in Lactating Dezhou Jennies

**Figure 1:**
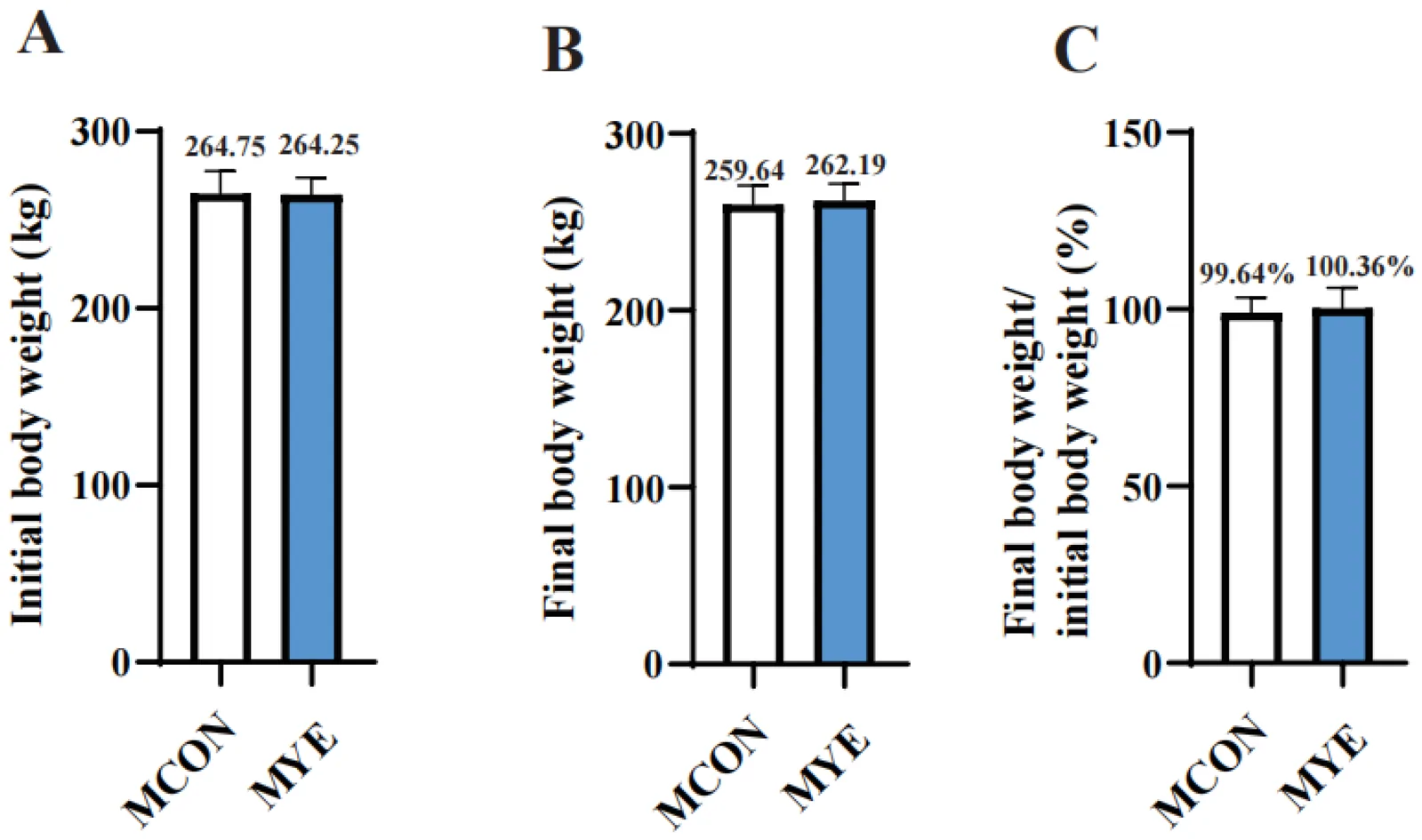
Effects of dietary yeast hydrolysate supplementation on body weight in lactating Dezhou jennies: (**A**) initial body weight; (**B**) final body weight; (**C**) body weight change during the experimental period. MCON, lactating Dezhou jennies fed the basal diet (*n* = 8); MYE, lactating Dezhou jennies fed the basal diet supplemented with yeast hydrolysate (*n* = 8).

As shown in Figure 1, the initial body weight did not differ between the MCON and MYE groups (*p* > 0.05), although the MYE group showed a slightly lower numerical value (Figure 1A). After the 60-day experimental period, the MYE group exhibited numerically greater final body weight and body weight change (final body weight/initial body weight) than the MCON group (Figure 1B,C). However, neither variable differed significantly between the two groups. Therefore, under the conditions of the present study, dietary yeast hydrolysate supplementation did not significantly affect body weight or body weight change in lactating Dezhou jennies.

### 3.2. Effects of Dietary YH Supplementation on Serum Biochemical Parameters in Lactating Dezhou Jennies

**Figure 2:**
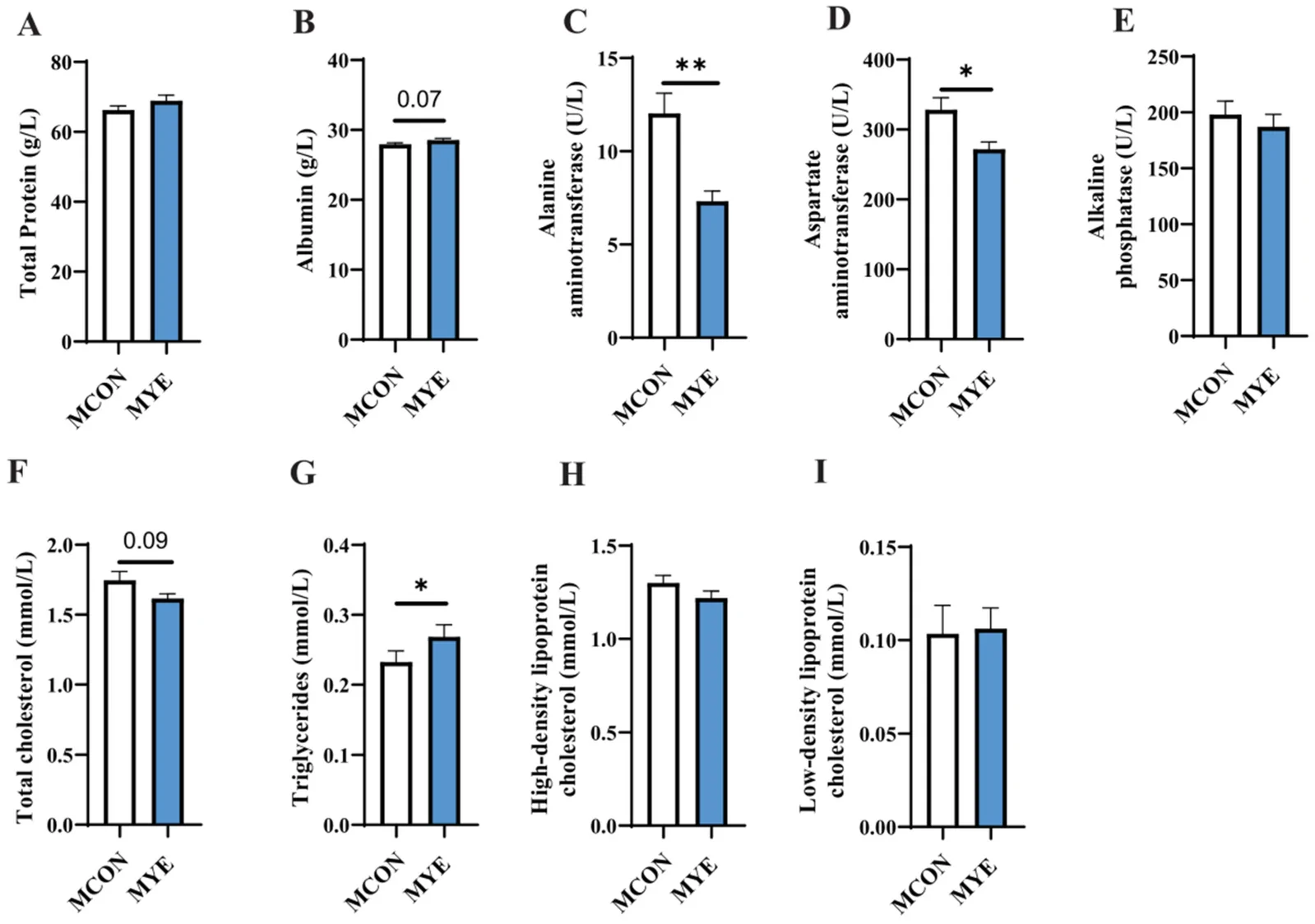
Effects of dietary yeast hydrolysate supplementation on serum biochemical parameters in lactating Dezhou jennies: (**A**) total protein (TP); (**B**) albumin (ALB); (**C**) alanine aminotransferase (ALT); (**D**) aspartate aminotransferase (AST); (**E**) alkaline phosphatase (ALP); (**F**) total cholesterol (TC); (**G**) triglyceride (TG); (**H**) high-density lipoprotein cholesterol (HDL-C); (**I**) low-density lipoprotein cholesterol (LDL-C). MCON, lactating Dezhou jennies fed the basal diet (*n* = 8); MYE, lactating Dezhou jennies fed the basal diet supplemented with yeast hydrolysate (*n* = 8). * *p* < 0.05, ** *p* < 0.01.

Serum biochemical parameters were measured to evaluate the physiological and metabolic status of lactating Dezhou jennies in response to dietary yeast hydrolysate supplementation. Compared with the MCON group, the MYE group had lower serum alanine aminotransferase (ALT) activity (*p* < 0.01; Figure 2C) and aspartate aminotransferase (AST) activity (*p* < 0.05; Figure 2D), whereas serum triglyceride (TG) concentration was higher (*p* < 0.05; Figure 2G). No significant differences were observed between the two groups in total protein (TP), albumin (ALB; *p* = 0.07), alkaline phosphatase (ALP), total cholesterol (TC; *p* = 0.09), high-density lipoprotein cholesterol (HDL-C), or low-density lipoprotein cholesterol (LDL-C) concentrations (*p* > 0.05; Figure 2A,B,E,F,H,I). Although numerically higher ALB concentrations and lower TC concentrations were observed in the MYE group, these differences were not statistically significant and therefore should be interpreted with caution.

### 3.3. Dietary YH Supplementation Reshaped the Serum Metabolomic Profile of Lactating Dezhou Jennies

**Figure 3:**
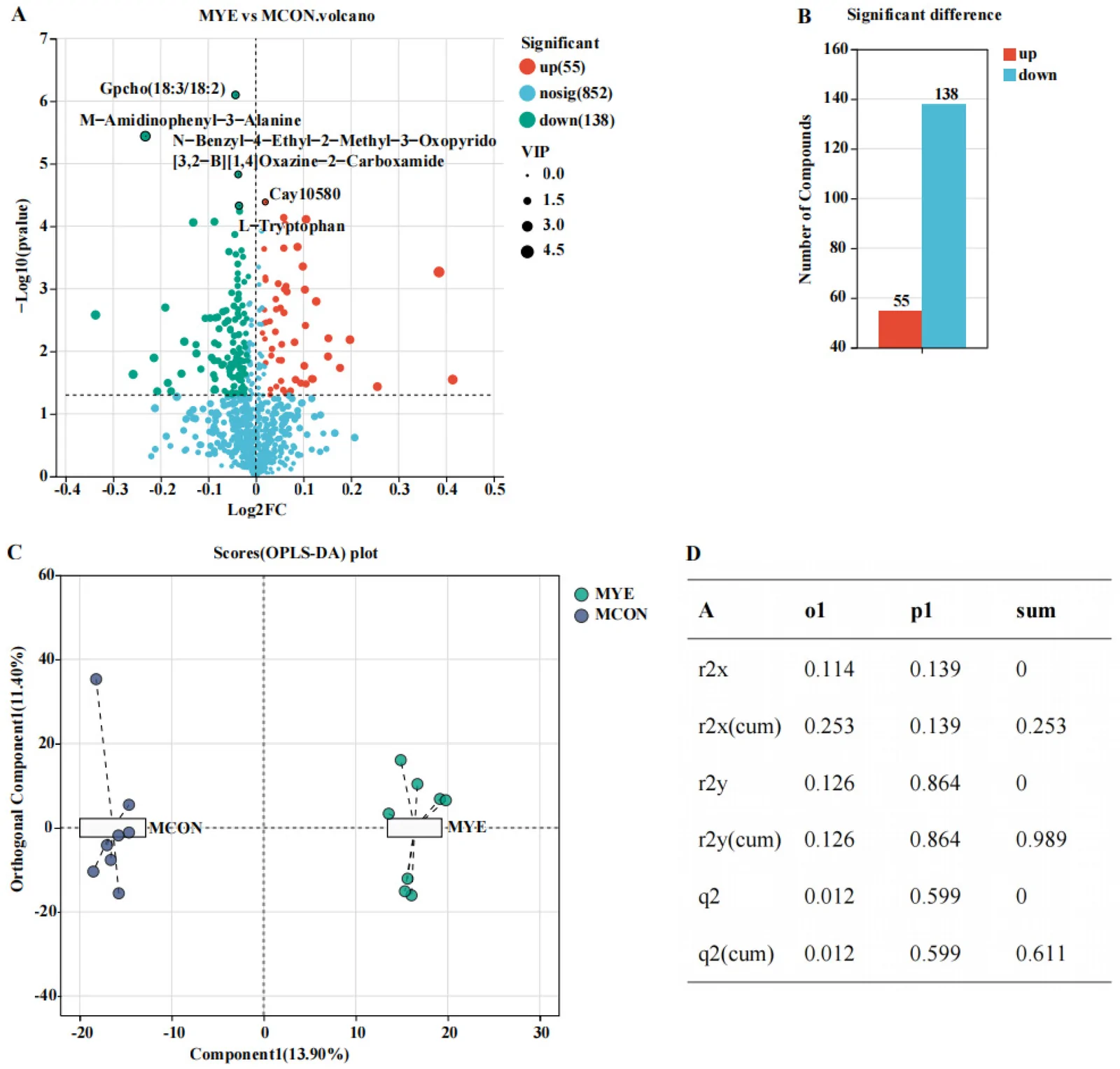
Effects of dietary yeast hydrolysate supplementation on the serum metabolomic profile of lactating Dezhou jennies: (**A**) volcano plot of differential metabolites between the MCON and MYE groups; (**B**) numbers of significantly upregulated and downregulated metabolites in the MYE group compared with the MCON group; (**C**) orthogonal partial least squares discriminant analysis (OPLS-DA) score plot based on serum metabolomic profiles; (**D**) summary of the OPLS-DA model parameters, including R^2^X, R^2^Y, and Q^2^ values for the predictive and orthogonal components. MCON, lactating Dezhou jennies fed the basal diet (*n* = 8); MYE, lactating Dezhou jennies fed the basal diet supplemented with yeast hydrolysate (*n* = 8).

Serum metabolomic profiling was performed to further characterize the metabolic responses of lactating Dezhou jennies to dietary yeast hydrolysate supplementation. Using the predefined exploratory screening criteria of a variable importance in projection (VIP) value > 1.0 and an unadjusted *p* value < 0.05, a total of 193 candidate differential metabolites were screened between the MCON and MYE groups, including 55 metabolites with relatively higher abundance and 138 metabolites with relatively lower abundance in the MYE group (Figure 3A,B). Because false discovery rate correction was not applied, these metabolites should be regarded as exploratory candidate features. The volcano plot showed that Cay10580 was among the representative metabolites with relatively higher abundance in the MYE group, whereas GpCho (18:3/18:2), L-tryptophan, M-amidinophenyl-3-alanine, and N-benzyl-4-ethyl-2-methyl-3-oxopyrido [3,2-b][1,4]oxazine-2-carboxamide showed relatively lower abundance (Figure 3A). In addition, the OPLS-DA score plot showed separation between the MCON and MYE groups (Figure 3C). The model yielded cumulative R^2^X, R^2^Y, and Q^2^ values of 0.253, 0.989, and 0.611, respectively, indicating a high goodness of fit for group classification and moderate predictive ability. The 200-permutation test further supported the validity of the model and indicated no evident overfitting (Figure 3D). Nevertheless, given the relatively small sample size, the OPLS-DA findings should be interpreted as exploratory evidence of differences in the overall serum metabolomic profiles between the two groups rather than definitive proof of treatment-induced metabolic alterations.

**Figure 4:**
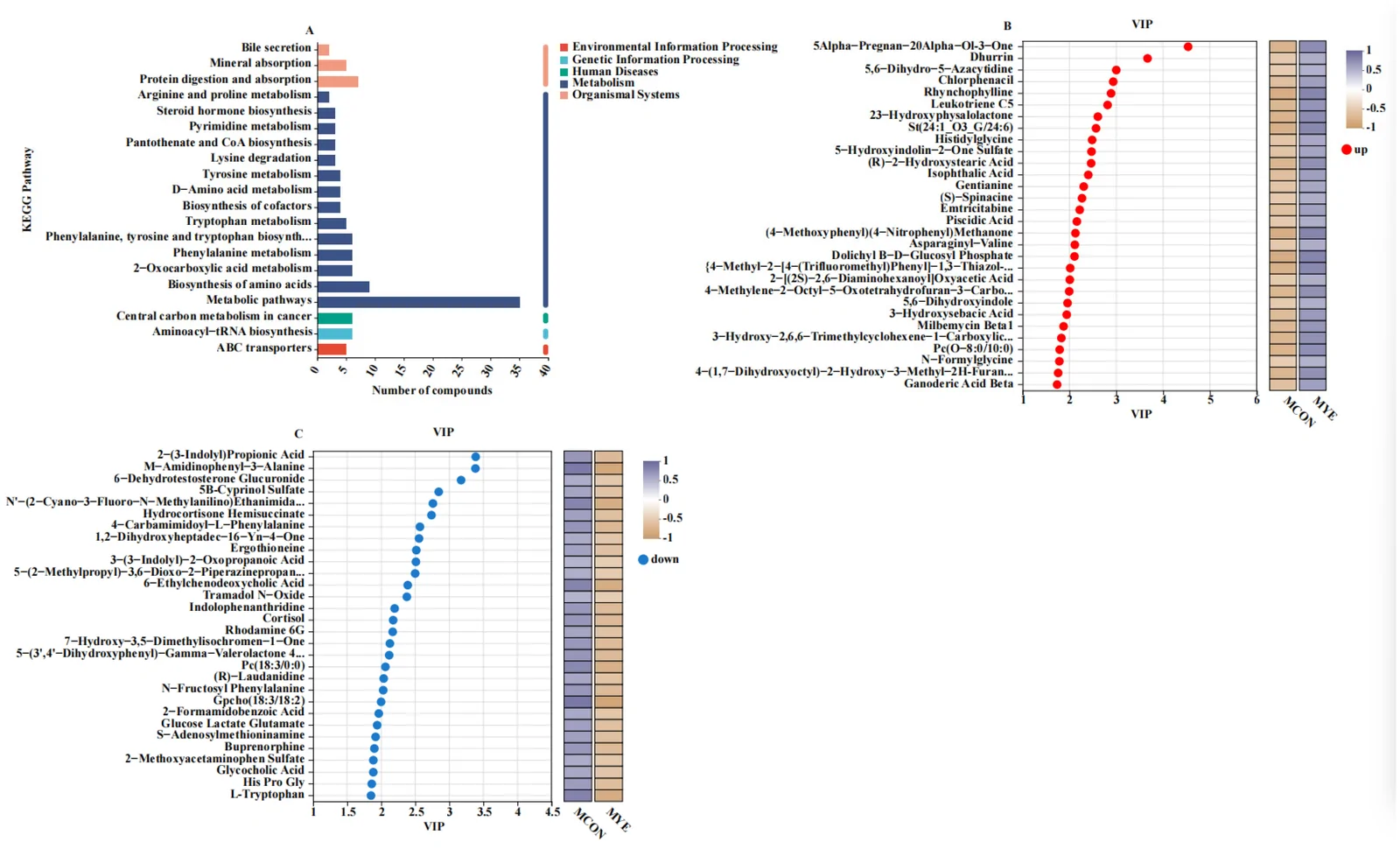
Effects of dietary yeast hydrolysate supplementation on serum metabolite profiles and KEGG pathway enrichment in lactating Dezhou jennies: (**A**) KEGG pathway classification of differential metabolites; (**B**) variable importance in projection (VIP) score plot of representative upregulated metabolites in the MYE group; (**C**) VIP score plot of representative downregulated metabolites in the MYE group. MCON, lactating Dezhou jennies fed the basal diet (*n* = 8); MYE, lactating Dezhou jennies fed the basal diet supplemented with yeast hydrolysate (*n* = 8).

KEGG annotation showed that the candidate differential metabolites were primarily mapped to metabolism-related pathways, including metabolic pathways, biosynthesis of amino acids, phenylalanine metabolism, 2-oxocarboxylic acid metabolism, and phenylalanine, tyrosine, and tryptophan biosynthesis (Figure 4A). Candidate differential metabolites were also mapped to pathways related to protein digestion and absorption, mineral absorption, bile secretion, and ABC transporters. These pathway annotations suggest that the candidate differential metabolites were associated with nutrient-related metabolic and transport pathways.

**Figure 5:**
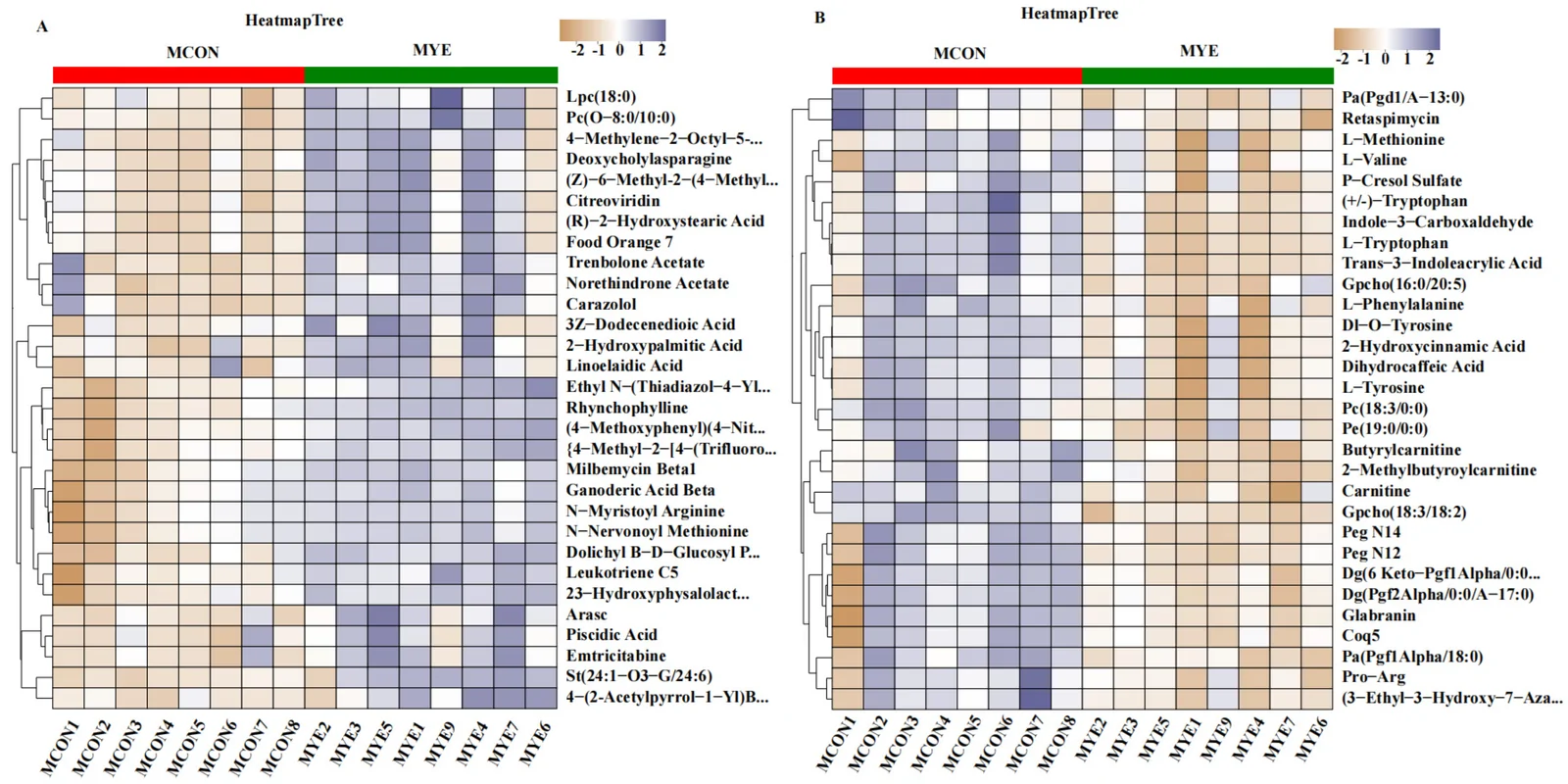
Effects of dietary yeast hydrolysate supplementation on serum metabolite profiles and KEGG pathway enrichment in lactating Dezhou jennies: (**A**) heatmap of representative upregulated metabolites; (**B**) heatmap of representative downregulated metabolites. MCON, lactating Dezhou jennies fed the basal diet (*n* = 8); MYE, lactating Dezhou jennies fed the basal diet supplemented with yeast hydrolysate (*n* = 8).

**Figure 6:**
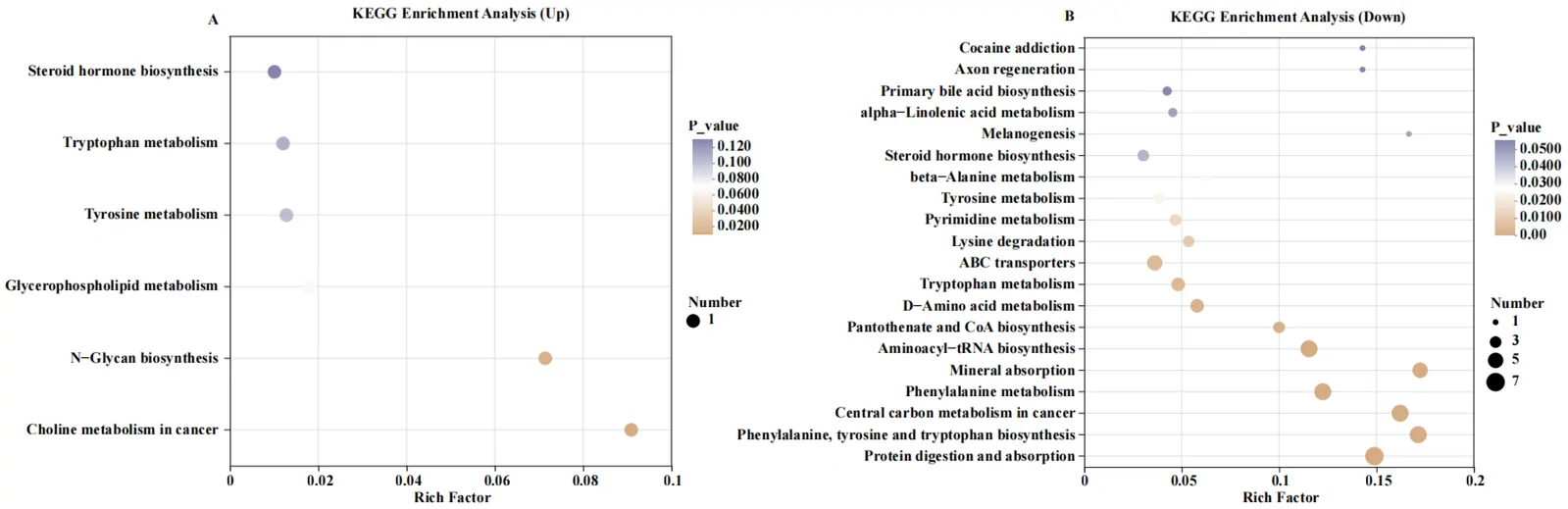
Effects of dietary yeast hydrolysate supplementation on serum metabolite profiles and KEGG pathway enrichment in lactating Dezhou jennies: (**A**) KEGG enrichment analysis of upregulated metabolites; (**B**) KEGG enrichment analysis of downregulated metabolites. MCON, lactating Dezhou jennies fed the basal diet (*n* = 8); MYE, lactating Dezhou jennies fed the basal diet supplemented with yeast hydrolysate (*n* = 8).

VIP analysis highlighted several putatively annotated metabolites that contributed to group discrimination. Among the metabolites with higher relative abundance in the MYE group, 5α-pregnan-20α-ol-3-one, Dhurrin, 5,6-dihydro-5-azacytidine, Rhynchophylline, and Leukotriene C5 showed relatively high VIP values (Figure 4B). In contrast, 2-(3-indolyl)propionic acid, M-amidinophenyl-3-alanine, 6-dehydrotestosterone glucuronide, cholic acid, and N-(2-cyano-3-furan-N-methylamino)ethanimidate were among the metabolites with lower relative abundance (Figure 4C). Hierarchical clustering further visualized differences in the relative abundance patterns of selected metabolites between the two groups (Figure 5A,B). KEGG enrichment analysis performed separately for metabolites with relatively higher and lower abundance in the MYE group identified several significantly enriched pathways. Metabolites with relatively higher abundance were mainly enriched in steroid hormone biosynthesis, tryptophan metabolism, tyrosine metabolism, glycerophospholipid metabolism, N-glycan biosynthesis, and choline metabolism in cancer (Figure 6A). Metabolites with relatively lower abundance were primarily enriched in protein digestion and absorption; phenylalanine, tyrosine, and tryptophan biosynthesis; phenylalanine metabolism; mineral absorption; aminoacyl-tRNA biosynthesis; pantothenate and CoA biosynthesis; D-amino acid metabolism; ABC transporters; lysine degradation; pyrimidine metabolism; steroid hormone biosynthesis; and primary bile acid biosynthesis (Figure 6B). Overall, these enrichment results indicate that the candidate differential metabolites identified in the exploratory metabolomic analysis were associated with pathways related to amino acid metabolism, lipid metabolism, bile acid metabolism, and nutrient transport.

### 3.4. Effects of Dietary YH Supplementation on Fecal Microbial Alpha Diversity in Lactating Dezhou Jennies

**Figure 7:**
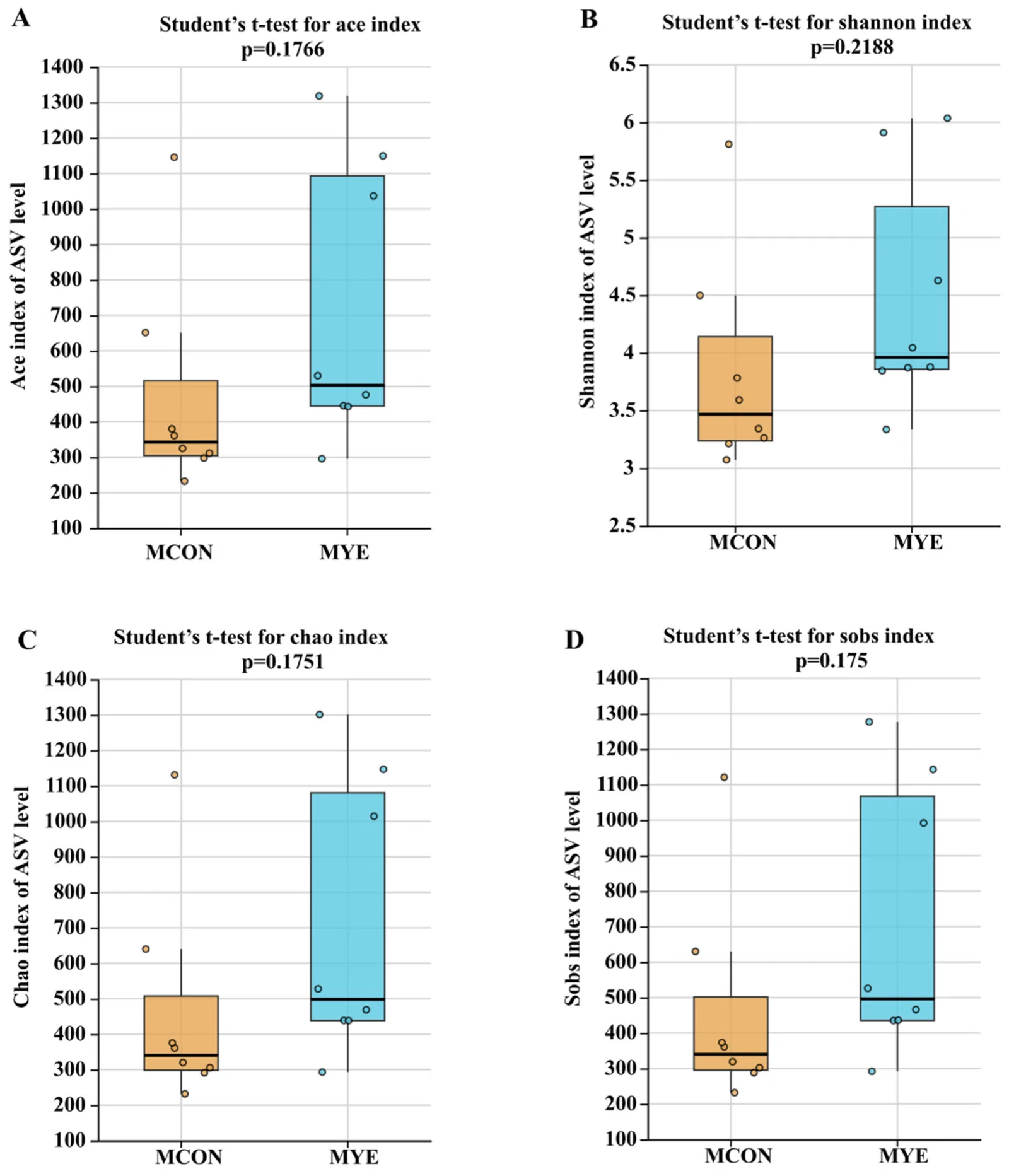
Effects of dietary yeast hydrolysate supplementation on the α-diversity of fecal microbiota in lactating Dezhou jennies: (**A**) ACE index; (**B**) Shannon index; (**C**) Chao1 index; (**D**) Sobs index. MCON, lactating Dezhou jennies fed the basal diet (*n* = 8); MYE, lactating Dezhou jennies fed the basal diet supplemented with yeast hydrolysate (*n* = 8).

Fecal microbial α-diversity was assessed to evaluate the effects of dietary YH supplementation on microbial richness and diversity in lactating Dezhou jennies. As shown in Figure 7, no significant differences were observed between the MCON and MYE groups in the ACE index (*p* = 0.1766; Figure 7A), Shannon index (*p* = 0.2188; Figure 7B), Chao1 index (*p* = 0.1751; Figure 7C), or Sobs index (*p* = 0.1750; Figure 7D). Although all four indices were numerically higher in the MYE group, these differences were not statistically significant. Therefore, dietary yeast hydrolysate supplementation did not significantly affect fecal microbial α-diversity in lactating Dezhou jennies.

### 3.5. Dietary YH Supplementation Modulated Specific Fecal Microbial Taxa in Lactating Dezhou Jennies

**Figure 8:**
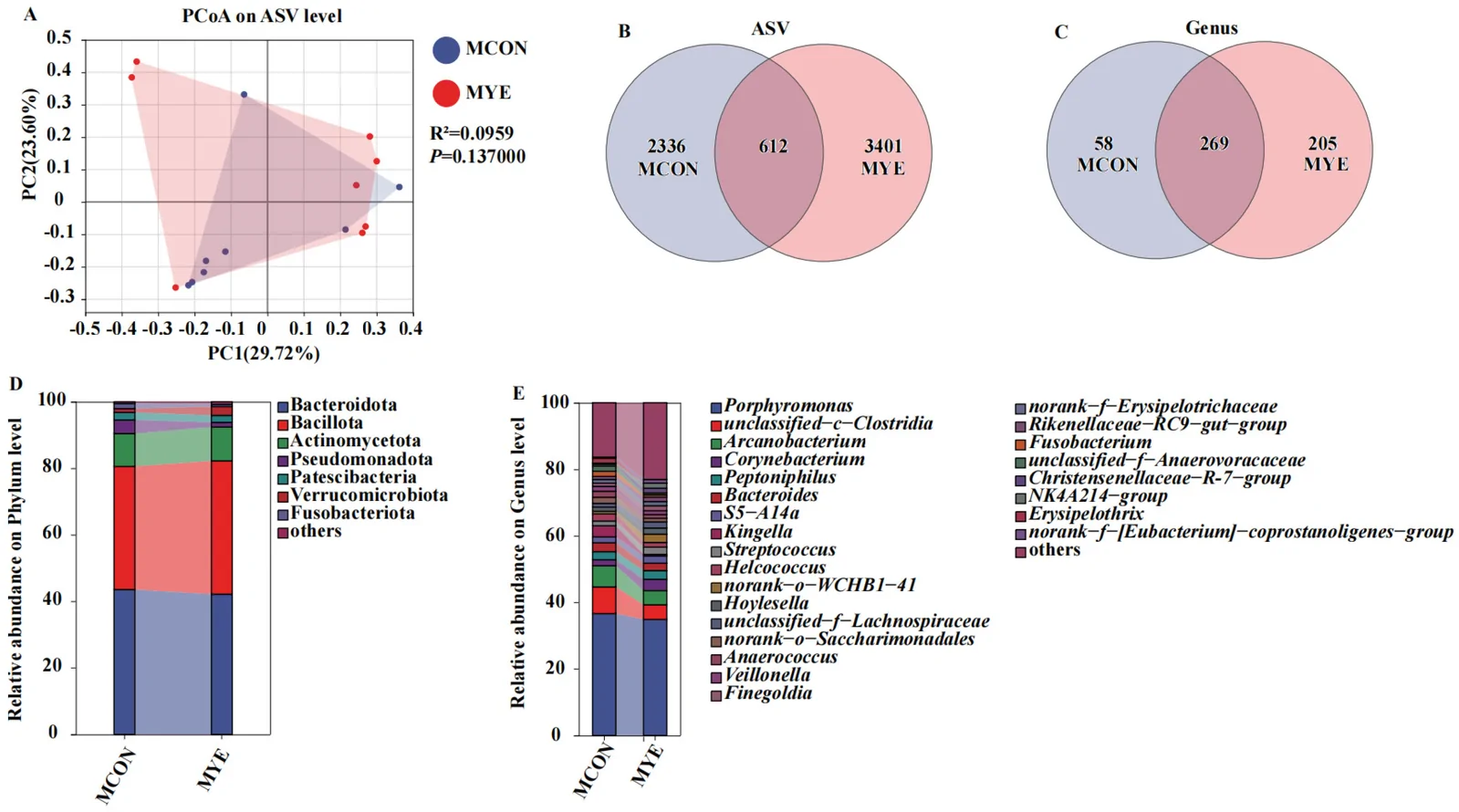
Effects of dietary yeast hydrolysate supplementation on fecal microbial composition in lactating Dezhou jennies: (**A**) principal coordinate analysis (PCoA) based on Bray–Curtis distances at the ASV level; (**B**) Venn diagram showing shared and unique ASVs between the MCON and MYE groups; (**C**) Venn diagram showing shared and unique genera between the MCON and MYE groups; (**D**) relative abundance of fecal microbiota at the phylum level; (**E**) relative abundance of fecal microbiota at the genus level. MCON, lactating Dezhou jennies fed the basal diet (*n* = 8); MYE, lactating Dezhou jennies fed the basal diet supplemented with yeast hydrolysate (*n* = 8).

**Figure 9:**
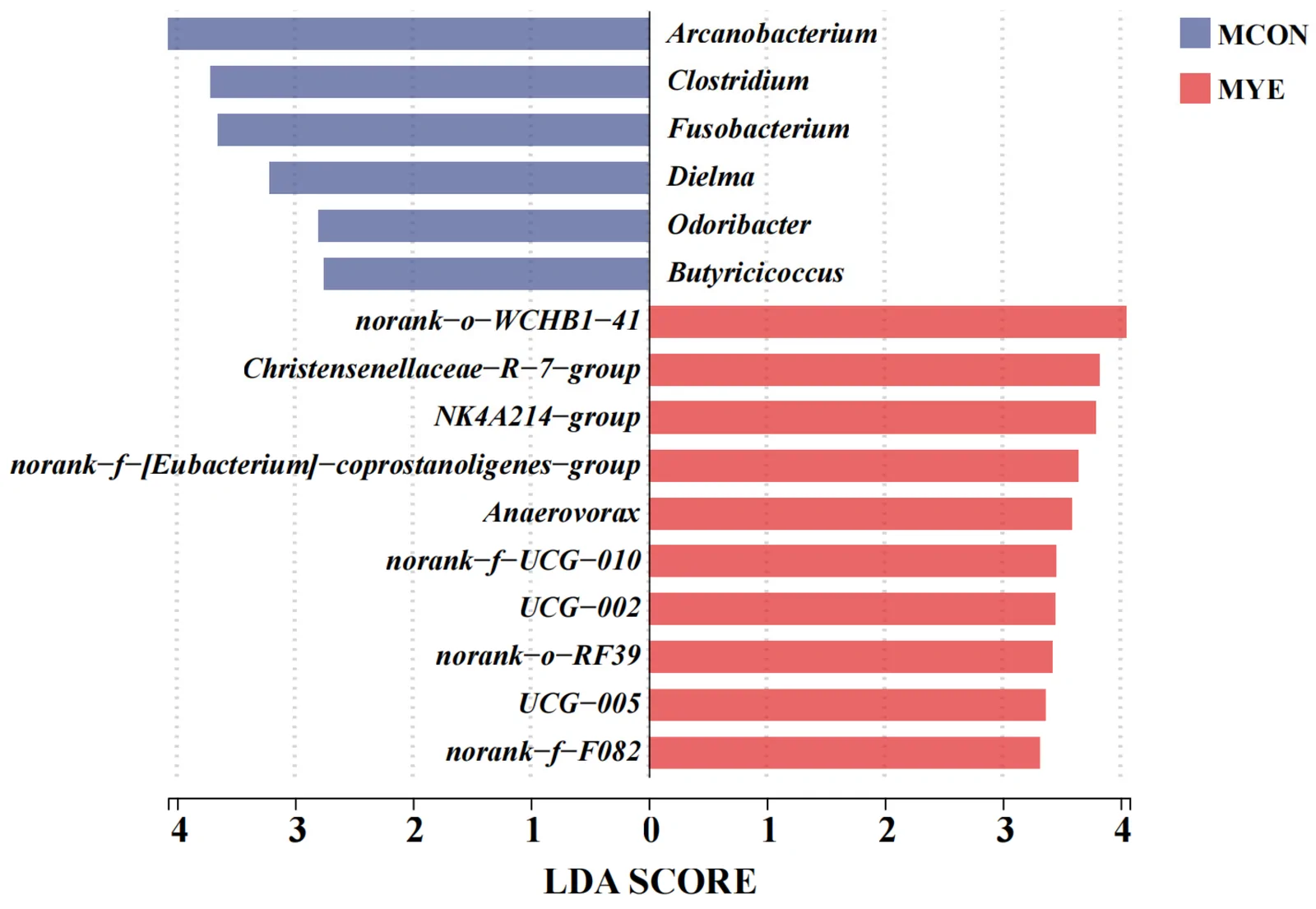
Effects of dietary yeast hydrolysate supplementation on differentially abundant fecal bacterial taxa in lactating Dezhou jennies. Linear discriminant analysis effect size (LEfSe) analysis showing bacterial taxa with differential relative abundances between the MCON and MYE groups. MCON, lactating Dezhou jennies fed the basal diet (*n* = 8); MYE, lactating Dezhou jennies fed the basal diet supplemented with yeast hydrolysate (*n* = 8).

Principal coordinate analysis (PCoA) based on Bray–Curtis distances at the ASV level was performed to evaluate fecal microbial community structure. PC1 and PC2 explained 29.72% and 23.60% of the total variation, respectively (Figure 8A). Although samples from the MCON and MYE groups showed partial separation in the ordination space, PERMANOVA analysis revealed no significant difference in overall fecal microbial community structure between the two groups (R^2^ = 0.0959, *p* = 0.137). Thus, dietary yeast hydrolysate supplementation did not induce a significant global shift in fecal microbial community composition. Venn diagram analysis based on the rarefied ASV table showed that 612 ASVs were shared between the two groups, whereas 2336 and 3401 ASVs were detected only in the MCON and MYE groups, respectively (Figure 8B). At the genus level, 269 genera were shared between the two groups, whereas 58 and 205 genera were detected only in the MCON and MYE groups, respectively (Figure 8C). These results suggest that the MYE group harbored a greater number of unique microbial features. At the phylum level, the fecal microbiota of both groups was dominated by Bacteroidota and Bacillota, followed by Actinomycetota, Pseudomonadota, Patescibacteria, Verrucomicrobiota, Fusobacteriota, and other minor phyla (Figure 8D). Compared with the MCON group, the MYE group showed a numerically lower relative abundance of Bacteroidota and a numerically higher relative abundance of Bacillota at the phylum level (Figure 8D). At the genus level, *Porphyromonas*, *unclassified Clostridia*, *Arcanobacterium*, *Corynebacterium*, *Peptoniphilus*, and *Bacteroides* were among the dominant taxa detected in the fecal samples (Figure 8E). LEfSe analysis identified several bacterial taxa that differed in relative abundance between the two groups. The MCON group was enriched in *Arcanobacterium*, *Clostridium*, *Fusobacterium*, *Dielma*, *Odoribacter*, and *Butyricicoccus*, whereas the MYE group showed higher relative abundances of *norank_o__WCHB1-41*, *Christensenellaceae_R-7_group*, *NK4A214_group*, *norank_f__[Eubacterium]_coprostanoligenes_group*, *Anaerovorax*, *norank_f__UCG-010*, *UCG-002*, *norank_o__RF39*, *UCG-005*, and *norank_f__F082* (Figure 9). Consistent with the non-significant PERMANOVA result, these findings indicate differences in the relative abundance of selected bacterial taxa rather than a significant overall shift in fecal microbial community composition. Overall, dietary YH supplementation was associated with differences in the relative abundance of selected fecal bacterial taxa, whereas the overall fecal microbial community structure remained unchanged.

## 4. Discussion

Yeast hydrolysate is a yeast-derived functional feed ingredient rich in bioactive peptides, amino acids, nucleotides, β-glucans, and mannan oligosaccharides, which have been associated with improved nutrient utilization, immune regulation, intestinal homeostasis, and productive performance in livestock [9,10,11,16,17,18,19]. Although YH is not a live probiotic, its yeast cell-wall-derived components, especially β-glucans and mannan oligosaccharides, may exert prebiotic-like effects by interacting with the intestinal microbial ecosystem and host immune–metabolic networks. In the present study, dietary YH supplementation was used to evaluate its effects on body weight change, serum biochemical status, serum metabolic profiles, and fecal microbiota in lactating Dezhou jennies. After the 60-day experimental period, the MYE group showed numerically higher final body weight and body weight change than the MCON group; however, these differences were not statistically significant. Although growth-related responses to hydrolyzed yeast or yeast-derived products have been reported in other livestock species [12,13,14,15,19], the present study does not provide sufficient evidence to determine whether dietary YH supplementation affected body weight in lactating Dezhou jennies. Moreover, feed intake, nutrient digestibility, milk yield, and energy balance were not measured. Therefore, the observed numerical differences should be interpreted cautiously and require confirmation in larger studies incorporating these outcomes.

Serum biochemical parameters provide important information on the physiological and metabolic status of animals. In this study, dietary YH supplementation significantly reduced serum ALT and AST activities in lactating Dezhou jennies, while all measured biochemical parameters remained within physiological reference ranges. ALT and AST are commonly used indicators of hepatic metabolic status and tissue integrity [20]. Thus, the lower ALT and AST activities in the MYE group suggest that YH supplementation may contribute to a more favorable systemic biochemical profile and may help maintain liver metabolic homeostasis in lactating jennies. This finding is consistent with evidence showing that yeast supplementation can improve liver metabolic status in lactating Hu sheep [21]. In addition, serum albumin was numerically higher, and total cholesterol was numerically lower in the MYE group, whereas triglyceride concentration was significantly increased. However, the differences in albumin and total cholesterol were not statistically significant, and these biochemical changes alone do not establish alterations in protein synthesis, lipid mobilization, or energy metabolism. Therefore, the results indicate only that dietary YH supplementation was associated with changes in selected serum biochemical parameters, and their physiological significance requires further investigation.

Consistent with the observed differences in serum biochemical parameters, untargeted metabolomic profiling identified differences in the circulating metabolite profiles of lactating Dezhou jennies between the MCON and MYE groups. Using the predefined exploratory screening criteria, 193 candidate differential metabolites were identified, and the OPLS-DA model showed separation between the two groups. KEGG annotation and enrichment analyses indicated that these candidate metabolites were associated with pathways related to amino acid metabolism, lipid metabolism, bile acid metabolism, steroid hormone biosynthesis, N-glycan biosynthesis, glycerophospholipid metabolism, mineral absorption, ABC transporters, and protein digestion and absorption. These pathway annotations suggest potential associations with biological processes involved in nutrient digestion, absorption, transport, energy metabolism, membrane composition, and cellular metabolism [22,23,24,25,26,27,28]. However, KEGG enrichment analysis is intended to facilitate biological interpretation of metabolomic data and does not provide evidence of altered pathway activity or underlying regulatory mechanisms. Several candidate metabolites, including L-tryptophan, L-valine, L-methionine, L-tyrosine, carnitine, cholic acid, and glycocholic acid, differed between the two groups. According to previous studies, these metabolites have been associated with amino acid metabolism, lipid metabolism, bile acid metabolism, immune function, and metabolic homeostasis [22,23,24]. Nevertheless, the observed differences in metabolite abundance should be interpreted as exploratory associations and do not demonstrate that these biological processes or metabolic pathways were directly altered by dietary YH supplementation. Overall, the metabolomic analysis suggests that dietary YH supplementation was associated with differences in selected circulating metabolites and nutrient-related pathway annotations in lactating Dezhou jennies. Because the metabolomic analysis was exploratory, untargeted in nature, and not supported by multiple-testing correction, targeted metabolite quantification, or mechanistic validation, these findings should be interpreted cautiously. Further targeted metabolomic, molecular, and functional studies are required to determine whether the observed metabolite associations have biological or physiological significance.

Dietary YH supplementation was associated with differences in the relative abundance of selected fecal bacterial taxa in lactating Dezhou jennies. However, neither α-diversity nor the overall fecal microbial community structure differed significantly between the two groups, indicating that dietary YH supplementation did not induce a global shift in the fecal bacterial community. Although all α-diversity indices were numerically higher in the MYE group and a greater number of ASVs and genera were detected only in the MYE group, these observations should be interpreted cautiously because they were not accompanied by significant changes in overall community diversity or structure. Previous studies have reported that yeast-derived products can influence gut microbial communities in livestock [15,19]; however, the present findings support only selective taxonomic differences rather than broad microbiota modulation. At the phylum level, the MYE group showed a numerically lower relative abundance of Bacteroidota and a numerically higher relative abundance of Bacillota. Because these differences were descriptive and no functional analyses were performed, they should not be interpreted as evidence of altered microbial fermentation or metabolic activity. Likewise, although members of Bacillota have been reported to participate in complex carbohydrate degradation and nutrient metabolism in herbivorous animals [29,30], the present study did not assess these functions directly. At the genus level, LEfSe analysis identified several bacterial taxa that differed in relative abundance between the two groups. The MCON group was enriched in Arcanobacterium, Clostridium, Fusobacterium, Dielma, Odoribacter, and Butyricicoccus, whereas the MYE group showed higher relative abundances of Christensenellaceae_R-7_group, norank_f__[Eubacterium]_coprostanoligenes_group, Anaerovorax, and several UCG-related taxa. Previous studies have associated members of the Christensenellaceae lineage with host metabolic status, anti-inflammatory responses, and intestinal barrier function [31,32], while Eubacterium species have been linked to carbohydrate fermentation, short-chain fatty acid production, and cholesterol-related metabolism [33,34]. Nevertheless, because only fecal 16S rRNA gene sequencing was performed, these published functional characteristics cannot be directly attributed to the taxa identified in the present study. Therefore, the observed taxonomic differences should be regarded as exploratory associations rather than evidence of altered microbial function or host physiological regulation. Future studies integrating metagenomics, metabolomics of microbial products, or fermentation measurements will be required to determine whether these taxonomic differences have functional significance.

Taken together, dietary YH supplementation was associated with differences in selected serum biochemical parameters, circulating metabolite profiles, and the relative abundance of certain fecal bacterial taxa in lactating Dezhou jennies. The metabolomic findings showed exploratory associations with amino acid-, lipid-, and bile acid-related pathway annotations, whereas the microbiota analysis identified differences in selected taxa without significant changes in overall fecal microbial diversity or community structure. These parallel observations do not demonstrate coordinated regulation or causal interactions between host metabolism and the fecal microbiota. Although the MYE group showed a numerically greater body weight change, the difference was not statistically significant. Given the limited sample size and the exploratory nature of the omics analyses, the findings should be regarded as preliminary associations rather than evidence of physiological or production benefits. Further studies with larger sample sizes, longer feeding periods, direct measurements of lactation performance, and functional validation are needed to determine the biological significance and practical relevance of YH supplementation in lactating Dezhou jennies.

## 5. Conclusions

In conclusion, these preliminary findings indicate that dietary YH supplementation affected selected serum biochemical indices and serum metabolomic profiles and was associated with changes in the relative abundance of certain fecal bacterial taxa in lactating Dezhou jennies. However, overall fecal microbial diversity and community structure were not significantly altered. Further studies with larger sample sizes are needed to confirm these findings and evaluate their biological and practical significance.

## Figures and Tables

**Table 1:** Composition and nutritional level of experimental concentrate (dry-matter basis).

Ingredients	Content (%)	Nutrients
Corn	31.75	Dry matter
Middlings bran	12.00	Crude protein
Wheat flour middling	21.00	Ash
DDGS	5.00	Crude fiber
Wheat bran	18.00	Ether extract
Soybean meal	6.65	Calcium
NaCl	0.63	Phosphorus
CaCO_3_	3.72	Lysine
CaHPO_4_	0.25	
Premix ^1^	1.00	
Total	100.00	

^1^ Premix/kg: VA, 600 KIU; VD3, 125 KIU; VE, 3500 IU; Fe, 2 g; Cu, 800 mg; Zn, 6 g; Mn, 13 g; I, 95 mg; Se, 50 mg.

## Data Availability

The raw sequencing data generated in this study have been deposited in the NCBI Sequence Read Archive (SRA) under accession number PRJNA1478050. All other data supporting the findings of this study are included in the article. Additional information is available from the corresponding author upon reasonable request.

## Abbreviations

Abbreviation	Full Name
ABC	ATP-binding cassette
ACE	Abundance-based coverage estimator
ALB	Albumin
ALP	Alkaline phosphatase
ALT	Alanine aminotransferase
AST	Aspartate aminotransferase
ASVs	Amplicon sequence variants
DADA2	Divisive Amplicon Denoising Algorithm 2
DNA	Deoxyribonucleic acid
HDL-C	High-density lipoprotein cholesterol
KEGG	Kyoto Encyclopedia of Genes and Genomes
LC–MS/MS	Liquid chromatography–tandem mass spectrometry
LDA	Linear discriminant analysis
LDL-C	Low-density lipoprotein cholesterol
LEfSe	Linear discriminant analysis effect size
MCON	Control group fed the basal diet
MYE	Yeast hydrolysate supplementation group
OPLS-DA	Orthogonal partial least squares discriminant analysis
PCoA	Principal coordinate analysis
PCR	Polymerase chain reaction
PERMANOVA	Permutational multivariate analysis of variance
QIIME	Quantitative Insights into Microbial Ecology
RDP	Ribosomal Database Project
SD	Standard deviation
Sobs	Observed species
SPSS	Statistical Package for the Social Sciences
TC	Total cholesterol
TG	Triglyceride
TP	Total protein
UCG	Uncultured group
UHPLC	Ultra-high-performance liquid chromatography
VIP	Variable importance in projection
YH	Yeast hydrolysate
16S rRNA	16S ribosomal RNA
